# Comparative Proteomic Analysis of Grapevine Rootstock in Response to Waterlogging Stress

**DOI:** 10.3389/fpls.2021.749184

**Published:** 2021-10-29

**Authors:** Xicheng Wang, Lichun Yan, Bo Wang, Yaming Qian, Zhuangwei Wang, Weimin Wu

**Affiliations:** Institute of Pomology, Jiangsu Academy of Agricultural Sciences, Jiangsu Key Laboratory for Horticultural Crop Genetic Improvement, Nanjing, China

**Keywords:** grapevine, leaf, waterlogging stress, tandem mass tags, proteomic analysis

## Abstract

Waterlogging severely affects global agricultural production. Clarifying the regulatory mechanism of grapevine in response to waterlogging stress will help to improve the waterlogging tolerance of grapevine. In the present study, the physiological and proteomic responses of SO4 grapevine rootstock to different waterlogging tolerances were comparatively assayed. The results showed that the activities of SOD and POD first increased and then decreased, while the change trend of CAT and APX activities was the opposite. In addition, the MDA and H_2_O_2_ contents increased after waterlogging treatment, but the chlorophyll a and chlorophyll b contents decreased. A total of 5,578 grapevine proteins were identified by the use of the tandem mass tag (TMT) labeling technique. Among them, 214 (103 and 111 whose expression was upregulated and downregulated, respectively), 314 (129 and 185 whose expression was upregulated and downregulated, respectively), and 529 (248 and 281 whose expression was upregulated and downregulated, respectively) differentially expressed proteins (DEPs) were identified in T0d vs. T10d, T10d vs. T20d, and T0d vs. T20d comparison groups, respectively. Enrichment analysis showed that these DEPs were mainly involved in glutathione metabolism, carbon fixation, amino sugar and nucleotide sugar metabolism, biosynthesis of amino acids, photosynthesis, carbon metabolism, starch, and sucrose metabolism, galactose metabolism, protein processing and ribosomes. To further verify the proteomic data, the expression of corresponding genes that encode eight DEPs was confirmed by quantitative reverse transcriptase-polymerase chain reaction (qRT-PCR). The results of this study presented an important step toward understanding the resistance mechanisms of grapevine in response to waterlogging stress at the proteome level.

## Introduction

Waterlogging is one of the major abiotic stresses that subjects plants to low-oxygen conditions and has a substantial negative influence on plant growth and development (Sairam et al., 2009). As an abiotic stress, waterlogging causes severe harm to nearly 16% of agricultural production areas worldwide, and the reasons for this harm include excessive rainfall and poor soil drainage (Ahsan et al., 2007; Xu et al., 2014; Wang et al., 2017; Sundgren et al., 2018). Remarkably, however, the area subjected to waterlogging is increasing in size every year (Zhou et al., 2020). Because of the low diffusion coefficient of oxygen in water, waterlogged tissues cannot obtain enough oxygen for aerobic respiration (Van Veen et al., 2014). The lack of oxygen severely affects plants at most growth and development stages and causes changes in energy-related metabolic pathways from aerobic respiration to anaerobic fermentation (Jackson and Colmer, 2005; Xu et al., 2014; Zhou et al., 2020). Anaerobic respiration can cause the accumulation of many toxic substances in plant tissues, such as ethanol and lactic acid (Tamang et al., 2014). Most plants are sensitive to waterlogging, and it is very difficult to obtain enough energy via conventional metabolic pathways when waterlogging is prolonged (Bailey-Serres and Voesenek, 2008; Van Veen et al., 2014). Therefore, to obtain enough energy, plants have evolved different ways to survive adverse living conditions (Shimamura et al., 2014). For example, rice and grass plants adapt to waterlogging stress via rapid stem/hypocotyl elongation (Evans, 2004; Jiang et al., 2010; Liu and Jiang, 2015).

Waterlogging stress results in a decrease in photosynthetic efficiency through stomatal closure and an increase in carbon dioxide diffusion resistance. Plants with strong resistance to waterlogging depend heavily on many morphological and physiological changes that are regulated by a variety of genes (Kadam et al., 2017; Luan et al., 2020). Through their signal transduction system, plants can perceive waterlogging stress and subsequently undergo a series of corresponding responses. In response to hypoxic stress, the expression of waterlogging-responsive genes is activated and, ultimately, changes in the levels of corresponding proteins occur (Xu et al., 2016; Yang et al., 2018). Further analysis has shown that waterlogging-responsive genes can be divided into those involved in function, transcriptional regulation and signal transduction (Zhou et al., 2020).

In recent years, great progress has been made in understanding the physiological regulatory mechanism underlying plant waterlogging tolerance. Currently, substantial progress has been made in recent applications of modern molecular biology techniques, including transcriptome and proteome sequencing techniques (Arora et al., 2017; Zhao et al., 2018; Cui et al., 2019). Furthermore, numerous waterlogging-responsive genes have also been identified by cDNA microarray techniques (Tang et al., 2005). Because changes in gene expression eventually result in changes in protein expression, proteomic approaches are widely used for comprehensive analyses of proteomic responses in waterlogging-stressed plants (Lin et al., 2016; Xu et al., 2016; Xu J. et al., 2018).

Grapevine (*Vitis vinifera* L.) is an economically important fruit species worldwide, and using rootstocks is very important for the improvement of grapevine stress resistance. Located on the western shore of the Pacific Ocean, most parts of China are dominated by a continental monsoonal climate, characterized by extreme weather events such as rainy summer–autumns. Heavy rains that often result in flooding or waterlogging are very detrimental to grapevines, especially during the rainy season, commonly called the plum rain season, which continues nearly 2 months during the late spring and early summer in the southern part of China. Grape production, wine quality and important flavor are very dependent upon climate. Heavy rains and bad climatic conditions affect grape growth and berry development. They are unfavorable for sugar accumulation, organic acid degradation and phenolic compound formation, which seriously hinder the further development of the table and wine grape industry in China (Zhu et al., 2018).

However, until now, only a few studies have focused on the waterlogging tolerance mechanism of grapevine (Li et al., 2013a, b; Zhu et al., 2018; Ruperti et al., 2019). Proteomics approaches represent effective tools for understanding the changes in protein expression levels in response to biotic and abiotic stresses in plants (Yang et al., 2014; Cui et al., 2015). However, proteomics has not been used to study the molecular mechanism underlying grapevine waterlogging tolerance. In recent years, tandem mass tag (TMT)-based quantitative proteomic approaches have been widely used for comparative analyses of proteomic changes (Guan et al., 2019; He et al., 2020). Therefore, to better understand the waterlogging resistance mechanism of grapevine plants, a TMT-based quantitative proteomic approach was used to analyze the specific roles of waterlogging stress on protein expression in the leaves of grapevine rootstocks.

These results will improve our understanding of the proteomic response of waterlogging stress-related proteins and will provide new insights into the physiological and molecular mechanisms associated with waterlogging stress in grapevine. Our findings also provide a list of potential candidates for further elucidating the molecular regulatory network underlying the response to waterlogging in other plants species.

## Materials and Methods

### Plant Materials and Experimental Design

One-year-old SO4 grapevine rootstock (*V*. *berlandieri* × *V*. *riparia*) plants were cultivated in the pots (25-cm diameter, 30-cm deep) in an artificial climate chamber at Jiangsu Academy of Agricultural Sciences, Nanjing, China (32°02’ N, 118°52’ E). The soil type used for grapevine growth comprised a mixture of peat, vermiculite, and perlite (3:2:1, v/v). Nine SO4 grapevine rootstock plants were grown in an artificial climate chamber (22°C for 12 h of light, 17°C for 12 h of darkness, photosynthetically active radiation (PAR) of 300 μmol/m^2^/s, and 70% relative humidity). Waterlogging stress was imposed by submerging the pots in plastic containers (90 cm length × 40 cm width × 40 cm height) with tap water kept at 2 cm above the top of the pots, and only the roots were in the water (Li et al., 2013b; Zhu et al., 2018). Three experimental treatments were imposed: waterlogging at the sixth-leaf stage for 0 days (T0d), 10 days (T10d), and 20 days (T20d). Three replicate middle functional leaves were sampled from each treatment. These samples were immediately frozen in liquid nitrogen and stored at −80°C for TMT analysis and RNA extraction.

### Physiological Indexes

The middle functional leaves from three replicate plants were sampled to assay the protective enzyme activity and the chlorophyll, MDA and H_2_O_2_ contents. The activities of SOD, POD, CAT, and APX were calculated using the methods described by Giannopolitis and Ries (1977), Hammerschmidt et al. (1982), Durner and Klessing (1996), and Wang and Jiang (2007), respectively. The chlorophyll, MDA and H_2_O_2_ contents were quantified according to the methods described by Du and Bramlage (1992), Wellburn (1994), and Bizzi et al. (2014), respectively. Three biological replicates were used in this experiment.

### Protein Extraction and Trypsin Digestion

Samples from grapevine rootstock leaves were put in a precooled mortar and ground into a fine powder in liquid nitrogen. Four volumes of lysis buffer were then added to the fine powder for protein extraction, followed by ultrasonication. Afterward, the samples were centrifuged at 20,000 g for 10 min at 4°C, and the supernatant was transferred to a clean tube. Subsequently, the protein was precipitated with precooled 20% TCA for 2 h at −20°C. The samples were then centrifuged at 12,000 g at 4°C for 10 min, after which the precipitate was collected and then washed with cold acetone three times. Finally, the protein was dissolved in 8 M urea, and the protein concentration was determined using a BCA kit protein assay (Bio-Rad, United States) according to the manufacturer’s instructions.

For digestion, the protein solution was reduced with 5 mM dithiothreitol for 30 min at 56°C and alkylated with 11 mM iodoacetamide for 15 min at room temperature in darkness. The protein sample was then diluted by the addition of 100 mM TEAB, with urea concentrations being less than 2 M. Finally, trypsin was added at a 1:50 trypsin:protein mass ratio for the first digestion overnight and a 1:100 trypsin:protein mass ratio for a second 4 h digestion.

### Tandem Mass Tag Labeling and High Performance Liquid Chromatography Fractionation

After trypsin digestion, the peptide was desalted through a Strata X C18 SPE column (Phenomenex) and vacuum dried. The peptides were reconstituted in 0.5 M TEAB and processed according to the manufacturer’s protocol for the TMT kit. Briefly, one unit of TMT reagent was thawed and reconstituted in acetonitrile. The peptide mixtures were then incubated for 2 h at room temperature and pooled, desalted and dried by vacuum centrifugation.

The tryptic peptides were fractionated by high-pH reversed-phase High Performance Liquid Chromatography using an Agilent 300 Extend C18 column (5 μm particles, 4.6 mm ID, 250 mm length). Briefly, the peptides were first separated with a gradient of 8–32% acetonitrile (pH 9.0) for more than 60 min into 60 fractions. The peptides were then combined into 18 fractions and dried by vacuum centrifugation.

### Liquid Chromatography-Tandem Mass Spectrometry Analysis

The tryptic peptides were dissolved in 0.1% formic acid (solvent A) and directly loaded onto a custom-made reversed-phase analytical column (15 cm length, 75 μm inner diameter). The gradient involved an increase from 6 to 23% solvent B (0.1% formic acid in 98% acetonitrile) for 26 min, followed by an increase from 23 to 35% for 8 min and then to 80% for 3 min; the solvent was then held at 80% for the last 3 min. All solvents were provided at a constant flow rate of 400 nL/min on an EASY-nLC 1000 UPLC system.

The peptides were subjected to an NSI source followed by tandem mass spectrometry (MS/MS) in a Q Exactive^TM^ Plus (Thermo Fisher Scientific) coupled to a UPLC instrument. The electrospray voltage applied was 2.0 kV. The m/z scan range was 350–1,800 for full scan, and intact peptides were detected by an Orbitrap device at a resolution of 70,000. Peptides were then selected for MS/MS using the NCE setting of 28, and the fragments were detected by the Orbitrap at a resolution of 17,500. The data-acquisition procedure alternated between one MS scan followed by 20 MS/MS scans, with 15.0 s dynamic exclusion. The automatic gain control (AGC) was set at 5E4, and the fixed first mass was set as 100 m/z.

### Database Searching

The resulting MS/MS data were processed using the MaxQuant search engine (v. 1.6.0.1) (Alexa and Rahnenfuhrer, 2010). The tandem mass spectra were searched against the data in the UniProt *Vitis vinifera* L. database concatenated with the reverse decoy database. Trypsin/P was specified as a cleavage enzyme allowing up to 2 missing cleavage sites. The mass tolerance for precursor ions was set to 20 ppm in the first search and 5 ppm in the main search, and the mass tolerance for fragment ions was set as 0.02 Da. Carbamidomethyl on Cys was specified as a fixed modification, and oxidation of Met was specified as a variable modification. The false discovery rate (FDR) was adjusted to < 1%, and the minimum score for peptides was set to > 40 to assess the confidence of the peptides (Ma et al., 2019). A TMT 10-plex kit was used for quantification of the resulting peptides. The quantitative level of peptides was determined according to its ion signal intensity ratio in the secondary spectrum. For differentially expressed proteins (DEPs), those with a fold change (FC) > 1.3 and an FDR < 0.05 were considered significantly differentially abundant.

### Venn Diagrams

Venny 2.1.0^1^ was used to determine the intersecting proteins among the differentially accumulated proteins whose expression was upregulated or downregulated.

### Protein Clustering

In thermographic clustering analysis, the quantitative information of the target protein set was normalized to a ± 1 interval. Second, Cluster 3.0 software^2^ was used to classify the two dimensions of the sample and protein expression simultaneously (distance algorithm, Euclidean; connection mode, Average linkage). Finally, Java Treeview software was used to generate hierarchical clustering thermograms.

### Bioinformatics Analysis

For Gene Ontology (GO) annotations, a reference proteome sequence was derived from the UniProt-GOA database.^3^ IDs of all identified proteins were converted to UniProt IDs, and the proteins were mapped to the reference proteome. The unmatched proteins were searched and annotated by InterProScan software (v5.13-52.0)^4^ with the sequence alignment method. All the proteins were grouped into three major categories: those associated with biological processes, cellular components and molecular functions.

For pathway annotation, the Kyoto Encyclopedia of Genes and Genomes (KEGG)^5^ online tool was used to describe each identified protein’s metabolic classification. All the identified proteins were mapped to the KEGG metabolic pathways by the KEGG online service software “KEGG mapper” and annotated by the KEGG online software “KAAS.”

### Enrichment Analysis

For the GO categories or KEGG pathways, two-tailed Fisher’s exact tests were performed to test the enrichment of the DEPs against all identified proteins. A GO or KEGG term with a *P*-value < 0.05 was considered significant.

### Protein-Protein Interaction Network

The gene symbols of the target proteins were first determined from their original databases. The gene symbol information was subsequently used to search the STRING database^6^ to identify direct and indirect interactions among the target proteins on the basis of the experimental evidence. Cytoscape software (version 3.2.1^7^) was used to construct the interaction network and to analyze it.

### RNA Extraction and Quantitative Real-Time PCR Validation

To validate the TMT data, eight DEPs were randomly selected for analysis of their corresponding gene transcript levels via qRT-PCR. Total RNA was extracted from grapevine rootstock leaves using a TRIzol kit according to the manufacturer’s protocol (Promega, Beijing, China). Residual contaminating DNA was removed by RNase-free DNase I (TaKaRa, Dalian, China). QRT-PCR was performed using a SYBR Premix Ex Taq Kit (TaKaRa, Dalian, China) and an ABI PRISM 7700 DNA Sequence Detection System (Applied Biosystems, Shanghai, China). The sequences of the primers used were designed using Primer Premier 5 software (Premier Biosoft International, Palo Alto, CA, United States). The *actin* gene (AB073011) was used as an internal standard to calculate relative fold differences based on comparative cycle threshold (2^–ΔΔCt^) values. Details of the primer sequences used in this study are presented in Supplementary Table 1. The qRT-PCR procedure was as follows: 1 μL of a 1/10 dilution of cDNA in H_2_O was added to 5 μL of 2 × SYBR^®^ Green buffer together with each primer at 0.1 μM, and H_2_O was added until the final volume reached 10 μL. The reactions were run in accordance with the following procedure: 50°C for 2 min; 95°C for 10 min; and then 40 cycles of 95°C for 30 s, 56°C for 30 s, and 72°C for 30 s. PCR was performed in 96-well optical reaction plates.

### Statistical Analysis

All physiological and qRT-PCR data analyses were performed using Excel and SPSS statistical software (version 19.0; SPSS Inc., Ltd., United States) via ANOVA followed by Tukey’s significant difference test at *p* ≤ 0.05. All the data are representative of three biological replicates.

## Results

### Reactive Oxygen Species Scavenging System

**FIGURE 1:**
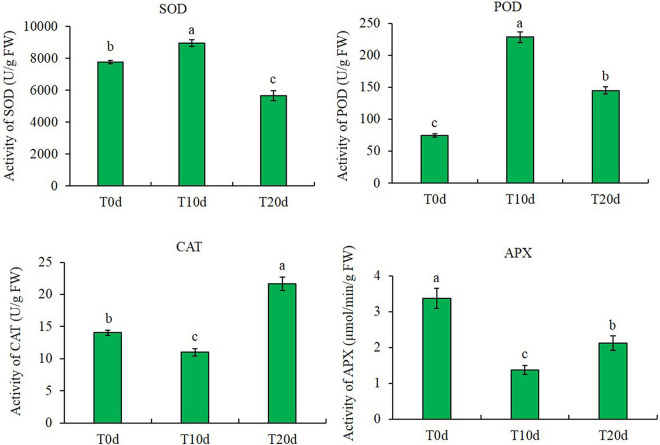
Changes in enzyme activity in different waterlogging treatments. Columns with the same letter were not significantly different at *p* < 0.05.

The results of the enzyme activity assay showed that the SOD and POD activities first increased and then decreased after waterlogging. In contrast, the CAT and APX activities tended to increase after decreasing. In addition, the activity of POD significantly increased after waterlogging treatment, and the activity of APX significantly decreased. However, compared to that in T0d (control, CK), the activity of SOD in T10d increased by 15.18% but decreased by 27.25% in T20d. The CAT activities decreased by 21.48% in T10d but increased by 54.29% in T20d compared to T0d (Figure 1).

### Malondialdehyde, H_2_O_2_ and Chlorophyll Contents

**FIGURE 2:**
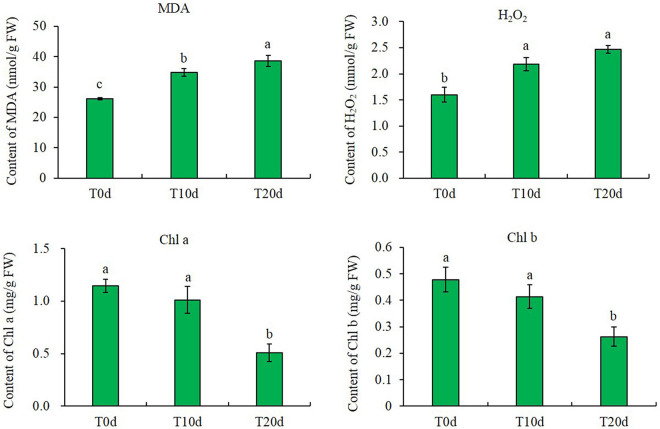
MDA, H_2_O_2_, and chlorophyll contents in different treatments. Columns with the same letter were not significantly different at *p* < 0.05.

The contents of MDA and H_2_O_2_ increased after waterlogging treatment. The MDA and H_2_O_2_ contents increased by approximately 32.87 and 36.55%, respectively, in T10d and 47.72 and 54.63%, respectively, in T20d compared to those in T0d. However, the chlorophyll content decreased after waterlogging treatment. The reductions in the contents of chlorophyll a and chlorophyll b were approximately 11.68 and 13.40%, respectively, in T10d and 55.58 and 45.09%, respectively, in T20d compared to those in T0d (Figure 2).

### Protein Profiles of Grapevine Rootstock Leaves Under Waterlogging Stress

**FIGURE 3:**
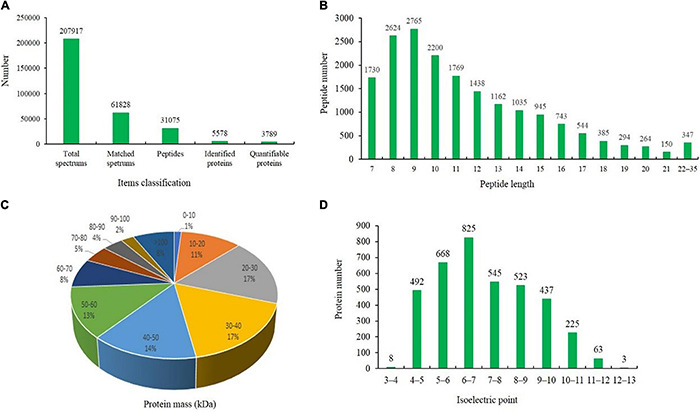
Results of the tandem mass tag (TMT)-based liquid chromatography-tandem mass spectrometry (LC-MS/MS) identification of the leaves of the grapevine rootstock. **(A)** Classification of the items used for identifying proteins. **(B)** Number and length of the identified peptides. **(C)** Distribution of the average molecular masses of identified proteins. **(D)** Distribution of the isoelectric points of the identified proteins.

Liquid chromatography-tandem mass spectrometry (LC-MS/MS) and TMT labeling were used to analyze the proteomic changes among different waterlogging-treated grapevine rootstock leaves. The expression profiles of the proteins extracted from grapevine rootstock leaves under T0d (labeled with 126, 127 N, and 127 C), T10d (labeled with 128 N, 128 C, and 129 N), and T20d (labeled with 129 C, 130 N, and 131) were analyzed. After quality validation, a total of 3,789 quantifiable proteins were identified from 31,075 peptides, which were matched to 61,828 spectra, with a false discovery rate of 1% (Figure 3A). The lengths of most identified peptides were 7–20 amino acid residues, suggesting that our sampling met the required standard (Figure 3B). The average molecular mass of the identified gene products ranged from 10 to 70 kDa, and the average mass error was < 0.02 Da, indicating a high mass accuracy of the MS data (Figure 3C). The distribution of the isoelectric points (pIs) of the identified proteins mainly ranged from 4.0 to 11.0, with most pIs ranging from 6.0 to 7.0 (Figure 3D). In addition, pairwise Pearson’s correlation coefficients displayed sufficient reproducibility in this experiment and reached the requirements for further study (Supplementary Figure 1).

### Identification of Differentially Expressed Proteins via Tandem Mass Tag

**FIGURE 4:**
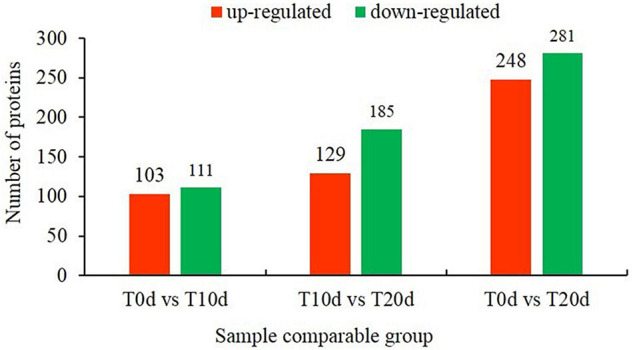
Number of differentially expressed proteins in the T0d vs. T10d, T10d vs. T20d, and T0d vs. T20d comparison groups.

According to the recognition criteria for DEPs (fold change > 1.3 and false discovery rate < 0.05), 214 (103 and 111 whose expression was upregulated and downregulated, respectively), 314 (129 and 185 whose expression was upregulated and downregulated, respectively) and 529 (248 and 281 whose expression was upregulated and downregulated, respectively) DEPs were identified in the T0d vs. T10d, T10d vs. T20d, and T0d vs. T20d comparison groups, respectively (Figure 4 and Supplementary Spreadsheet 1). To assess the DEPs among each group in detail, we identified the top 10 DEPs whose expression was upregulated and the top 10 downregulated DEPs according to their fold changes (Table 1). Further analysis showed that five, four, and two common top-10 DEPs among the T0d vs. T10d and T10d vs. T20d comparison groups, the T10d vs. T20d and T0d vs. T20d comparison groups and the T0d vs. T10d and T0d vs. T20d comparison groups were found, respectively, but only one common top DEP was found in all of the above three comparison groups.

**TABLE 1:** Top 10 differentially expressed proteins whose expression was upregulated and downregulated between groups.

**Groups**	**No.**	**Protein ID**	**Protein description**	**Fold change**	***P*-value**
T0d vs. T10d	Up-1	VIT_15s0048g02600	Granule-bound starch synthase 1	2.82	2.16988E-05
	Up-2	VIT_07s0005g01590	Proline-rich receptor-like protein kinase PERK15 isoform X2	2.72	1.56571E-05
	Up-3	VIT_14s0108g00590	ATP-dependent zinc metalloprotease FTSH 6	2.55	1.65282E-06
	Up-4	VIT_10s0116g00560	Polyphenol oxidase, chloroplastic	2.47	0.000758964
	Up-5	VIT_13s0019g00860	Heat shock protein, peroxisomal	2.38	0.000390993
	Up-6	VIT_16s0098g01060	Small heat shock protein, chloroplastic	2.17	0.003859804
	Up-7	VIT_14s0030g02190	Inactive ATP-dependent zinc metalloprotease FTSHI 5, chloroplastic	2.12	0.000147408
	Up-8	VIT_13s0019g03160	Heat shock protein	1.87	0.002806494
	Up-9	VIT_05s0049g01960	Uncharacterized protein LOC100256206	1.80	8.29011E-05
	Up-10	VIT_02s0025g04830	Cu/Zn-superoxide dismutase copper chaperone precursor	1.78	0.002496828
	Down-1	VIT_03s0063g02610	Glycine-rich RNA-binding protein GRP2A	0.34	7.00699E-06
	Down-2	VIT_04s0008g00640	Clathrin interactor EPSIN 1	0.54	0.000210712
	Down-3	VIT_17s0000g09680	Ribonucleoprotein, chloroplastic	0.54	0.000574883
	Down-4	VIT_00s0415g00040	Glycine-rich domain-containing protein 1	0.54	0.004856948
	Down-5	VIT_16s0022g01340	Cryptochrome DASH, chloroplastic/mitochondrial	0.55	0.00050511
	Down-6	VIT_03s0091g00160	Basic secretory protease	0.56	0.024218434
	Down-7	VIT_18s0001g03880	Calcium-binding protein CML29	0.58	0.004124814
	Down-8	VIT_00s0373g00010	Tubulin alpha chain isoform X2	0.60	0.016891712
	Down-9	VIT_05s0077g02190	Methylecgonone reductase-like isoform X1	0.61	0.012185056
	Down-10	VIT_01s0011g00830	Uncharacterized protein LOC100262861	0.61	0.023996651
T10d vs. T20d	Up-1	VIT_00s0415g00040	Glycine-rich domain-containing protein 1	3.93	7.47102E-08
	Up-2	VIT_02s0025g03760	Myosin-9 isoform X1	3.92	0.044866654
	Up-3	VIT_19s0135g00120	Cytochrome P450	3.42	0.001273289
	Up-4	VIT_13s0019g02490	Cucumisin	2.82	0.007288936
	Up-5	VIT_09s0002g03270	Disease resistance protein	2.55	0.006076873
	Up-6	VIT_02s0012g01380	Uncharacterized protein LOC100255790	2.39	0.000198999
	Up-7	VIT_02s0025g04540	SPX domain-containing membrane protein	2.05	8.83386E-06
	Up-8	VIT_03s0063g02610	Glycine-rich RNA-binding protein GRP2A	2.04	4.42583E-06
	Up-9	VIT_14s0006g01390	Pentatricopeptide repeat-containing protein	1.99	0.006123679
	Up-10	VIT_11s0037g00800	Serine carboxypeptidase II-2	0.97	1.962418537
	Down-1	VIT_19s0014g00160	Chlorophyll a-b binding protein of LHCII type 1	0.22	0.008554293
	Down-2	VIT_07s0005g01590	Proline-rich receptor-like protein kinase PERK15 isoform X2	0.29	3.07704E-06
	Down-3	VIT_13s0067g02270	Mediator of RNA polymerase II transcription subunit 15a-like isoform X7	0.31	0.016559313
	Down-4	VIT_12s0028g01390	Small heat shock protein	0.40	2.43018E-05
	Down-5	VIT_10s0116g00560	Polyphenol oxidase, chloroplastic	0.40	2.61439E-05
	Down-6	VIT_14s0068g01160	Light-regulated protein	0.41	0.000148996
	Down-7	VIT_13s0064g00670	Photosystem II 5 kDa protein, chloroplastic	0.43	0.002034664
	Down-8	VIT_08s0007g02290	Receptor protein kinase TMK1	0.45	7.4762E-05
	Down-9	VIT_05s0020g02510	Guanylate kinase 2, chloroplastic/mitochondrial	0.49	9.49831E-05
	Down-10	VIT_14s0108g00590	ATP-dependent zinc metalloprotease	0.50	8.75672E-05
T0d vs. T20d	Up-1	VIT_08s0007g08310	Alpha-galactosidase isoform X1	2.66	0.000305292
	Up-2	VIT_19s0135g00120	Cytochrome P450	2.66	9.59387E-06
	Up-3	VIT_06s0004g04700	Outer envelope pore protein 16, chloroplastic	2.43	1.51027E-05
	Up-4	VIT_13s0019g02180	Tropinone reductase homolog	2.31	2.78223E-05
	Up-5	VIT_05s0020g03140	Hexose transporter	2.30	7.88165E-06
	Up-6	VIT_07s0031g03210	Serine/threonine-protein kinase SRK2A	2.22	1.69898E-06
	Up-7	VIT_00s0415g00040	Glycine-rich domain-containing protein 1	2.12	0.001263125
	Up-8	VIT_14s0108g01560	Alpha-1,4 glucan phosphorylase L isozyme, chloroplastic/amyloplastic	2.09	3.77643E-06
	Up-9	VIT_11s0016g05770	Galactinol–sucrose galactosyltransferase 6	2.03	0.000186519
	Up-10	VIT_02s0025g02790	Granule-bound starch synthase 1, chloroplastic/amyloplastic	1.99	0.000256633
	Down-1	VIT_13s0019g02050	Ribulose bisphosphate carboxylase/oxygenase activase, chloroplastic	0.39	0.00051936
	Down-2	VIT_14s0068g01160	Light-regulated protein	0.42	0.000477278
	Down-3	VIT_14s0030g00660	Bifunctional 3-dehydroquinate dehydratase/shikimate dehydrogenase, chloroplastic	0.43	0.007307859
	Down-4	VIT_13s0064g00670	Photosystem II 5 kDa protein, chloroplastic	0.44	0.010635022
	Down-5	VIT_19s0090g01040	Uncharacterized protein LOC100257237	0.44	9.63759E-05
	Down-6	VIT_06s0004g02740	GATA transcription factor 8-like	0.44	9.68923E-05
	Down-7	VIT_05s0020g00600	1-Cys peroxiredoxin	0.45	8.21825E-06
	Down-8	VIT_13s0067g02560	Uncharacterized protein LOC100241651	0.45	0.000114853
	Down-9	VIT_16s0022g01340	Cryptochrome DASH, chloroplastic/mitochondrial	0.47	2.59881E-05
	Down-10	VIT_04s0008g07110	Aspartic protease in guard cell2	0.47	0.000790736

For the five common top DEPs between the T0d vs. T10d and T10d vs. T20d comparison groups, the expression of three DEPs (VIT_07s0005g01590, VIT_14s0108g00590 and VIT_10s0116g00560) was upregulated in the T0d vs. T10d group but downregulated in the T10d vs. T20d group. However, the expression of the other two DEPs (VIT_03s0063g02610 and VIT_00s0415g00040) was downregulated in the T0d vs. T10d group and upregulated in the T0d vs. T20d group. These DEPs included proline-rich receptor-like protein kinase PERK15 isoform X2, ATP-dependent zinc metalloprotease, polyphenol oxidase, glycine-rich RNA-binding protein GRP2A and glycine-rich domain-containing protein 1. In the T10d vs. T20d and T0d vs. T20d comparison groups, two common top DEPs (VIT_00s0415g00040 and VIT_19s0135g00120) showed consistent upregulated expression, but the other two common top DEPs (VIT_14s0068g01160 and VIT_13s0064g00670) showed consistent downregulated expression. These DEPs included glycine-rich domain-containing protein 1, cytochrome P450, a light-regulated protein and photosystem II 5 kDa protein. In the T0d vs. T10d and T0d vs. T20d groups, one DEP (VIT_16s0022g01340, hexose transporter) showed consistently downregulated expression, but the expression of the other DEP (VIT_00s0415g00040, glycine-rich domain-containing protein 1) was downregulated in the T0d vs. T10d group and upregulated in the T0d vs. T20d group. This showed that the expression level of the VIT_00s0415g00040 protein first decreased and then increased. Moreover, the VIT_00s0415g00040 protein was also the only common top DEP found among the three groups.

**FIGURE 5:**
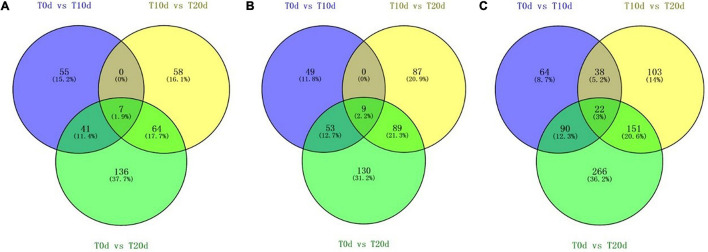
Venn diagram of DEPs whose expression was co-upregulated **(A)** and co-downregulated **(B)** and the total number of DEPs whose expression was co-upregulated or co-downregulated **(C)** in each experimental group comparison.

Additional large-scale analysis of DEPs between groups showing co-upregulated and co-downregulated expression was also carried out. From Figure 5, we found that seven DEPs showed co-upregulated expression, and nine DEPs showed co-downregulated expression among all three groups (Figures 5A,B). When the total number of proteins whose expression was co-upregulated and co-downregulated was counted, 22 DEPs common to all three groups were found (Figure 5C). This suggests that the 22 common DEPs, especially 7 whose expression was co-upregulated and 9 whose expression was co-downregulated, may play an important role in the response to waterlogging stress in grapevine.

### Cluster Analysis

**FIGURE 6:**
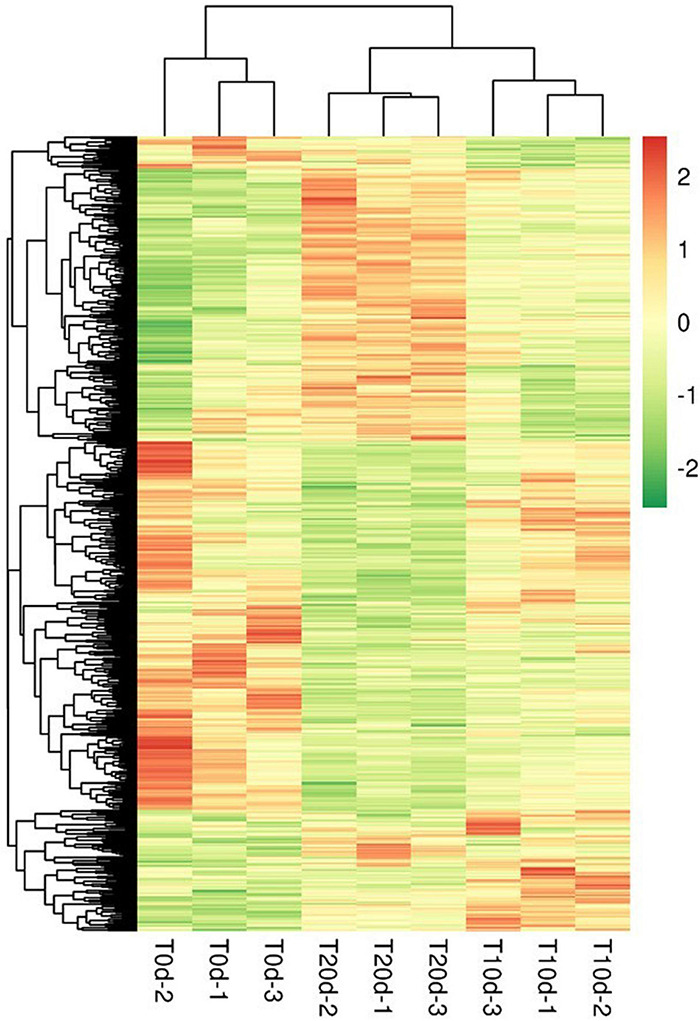
Cluster analysis of differentially expressed proteins. Through a horizontal comparison, the samples could be classified into three categories, suggesting that the selected DEPs could effectively be distinguished among the samples. A vertical comparison indicated that proteins could be classified into two categories with opposite directional variation, demonstrating the rationality of the selected DEPs.

The hierarchical clustering results were expressed as a respective heat map (Figure 6). By a horizontal comparison, the samples could be classified into three categories: T0d, T10d, and T20d. This suggests that the selected DEPs could be effectively distinguished among samples with a high degree of accuracy. Through a vertical comparison, the selected proteins could be classified into two categories with opposite directional variation, which clustered the expression patterns of DEPs into three groups (Supplementary Spreadsheet 2), demonstrating the rationality of the selected DEPs. The cluster analysis thus supported that the DEPs screened through our experiment were accurate.

### Gene Ontology Analysis of Differentially Expressed Proteins

To further understand the nature of the identified DEPs, we annotated their functions and features using GO functional enrichment analysis. The DEPs (214, 314, and 529) corresponded to 196, 262, and 412 GO terms in the T0d vs. T10d, T10d vs. T20d, and T0d vs. T20d comparison groups, respectively. Among these GO terms, there were 46 molecular function terms, 113 biological process terms and 37 cellular component terms in the T0d vs. T10d group. The GO terms in the T10d vs. T20d group included 69 molecular function terms, 142 biological process terms, and 51 cellular component terms, and the GO terms in the T0d vs. T20d group included 124 molecular function terms, 235 biological process terms and 53 cellular component terms (Supplementary Spreadsheet 3).

**FIGURE 7:**
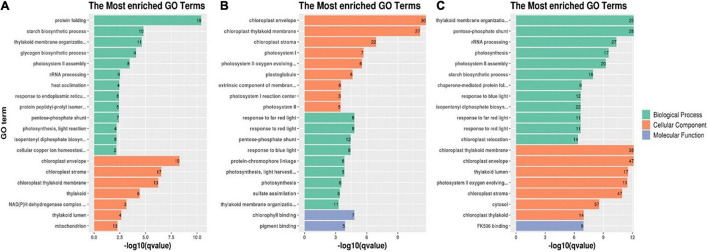
Twenty most enriched GO terms of DEPs in the T0d vs. T10d **(A)**, T10d vs. T20d **(B)**, and T0d vs. T20d groups **(C)**. For details of the GO enrichment analysis of DEPs, see Supplementary Spreadsheet 4 and Supplementary Figures 2–4.

These GO terms were classified on the basis of their enrichment to investigate the properties of the DEPs in each group. The 20 most enriched GO terms in each group and their association with the three main GO categories are presented in Figure 7. As shown in Figure 7A, for the T0d vs. T10d comparison group, the 20 most enriched GO terms for DEPs included 13 biological process terms (protein folding, starch biosynthetic process, thylakoid membrane organization, the glycogen biosynthetic process, photosystem II assembly, rRNA processing, heat acclimation, response to endoplasmic reticulum stress, protein peptidyl-prolyl isomerization, the pentose-phosphate shunt, photosynthesis, the isopentenyl diphosphate biosynthetic process, and cellular copper ion homeostasis) and 7 cellular component terms (the chloroplast envelope, chloroplast stroma, chloroplast thylakoid membrane, thylakoid, NAD(P)H dehydrogenase complex, thylakoid lumen, and mitochondrion). However, no GO terms associated with molecular functions were detected for the 20 most enriched GO terms.

Among molecular function terms in the T10d vs. T20d group, chlorophyll binding was the most prevalent term for DEPs. Response to far-red light, response to red light, the pentose-phosphate shunt, response to blue light, protein-chromophore linkage, light harvesting in photosystem I, photosynthesis, sulfate assimilation, and thylakoid membrane organization were the nine top terms for DEPs associated with biological processes. The most abundant GO terms for DEPs associated with cellular components were chloroplast envelope, chloroplast thylakoid membrane, chloroplast stroma, photosystem I, photosystem II oxygen-evolving complex, plastoglobule, extrinsic component of membranes, photosystem I reaction center, and photosystem II (Figure 7B).

With respect to GO enrichment of DEPs in the T0d vs. T20d group, FK506 binding was the most enriched term associated with molecular function, and it was also the only enriched GO term associated with molecular function. For the other DEPs, thylakoid membrane organization, the pentose-phosphate shunt and rRNA processing were the top three terms associated with biological processes, and chloroplast thylakoid membrane, chloroplast envelope and thylakoid lumen were the three most enriched terms associated with cellular components (Figure 7C).

### Kyoto Encyclopedia of Genes and Genomes Pathway Analysis of Differentially Expressed Proteins

**FIGURE 8:**
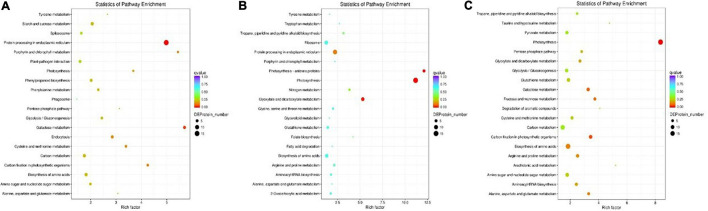
KEGG pathway enrichment analysis of DEPs in the T0d vs. T10d **(A)**, T10d vs. T20d **(B)**, and T0d vs. T20d **(C)** groups. For details of the KEGG pathway analysis of the DEPs, see Supplementary Spreadsheet 5.

To identify the biological pathways operating during waterlogging of grapevine, we mapped the DEPs in each group to reference pathways whose information is housed in the KEGG pathway database (see text footnote 5). Among the DEPs identified in each group, 65 DEPs, 107 DEPs, and 181 DEPs had a KEGG Orthology (KO) ID and were mapped to 23 pathways, 38 pathways, 62 pathways in the T0d vs. T10d, T10d vs. T20d, and T0d vs. T20d comparison groups, respectively (Supplementary Spreadsheet 5). The enrichment results of the top 20 KEGG pathways in each group are presented in Figure 8. As shown in Figure 8A, protein processing in the endoplasmic reticulum (15 DEPs), galactose metabolism (5 DEPs), carbon fixation in photosynthetic organisms (4 DEPs), porphyrin and chlorophyll metabolism (3 DEPs) and cysteine, and methionine metabolism (4 DEPs) were highly enriched in the T0d vs. T10d group (Figure 8A). For the T10d vs. T20d group, photosynthesis (15 DEPs), photosynthesis-antenna proteins (5 DEPs), glyoxylate, and dicarboxylate metabolism (7 DEPs), protein processing in the endoplasmic reticulum (11 DEPs), and nitrogen metabolism (3 DEPs) showed high enrichment (Figure 8B). In the T0d vs. T20d group, the DEPs were mostly involved in the following pathways: photosynthesis (19 DEPs); carbon fixation in photosynthetic organisms (9 DEPs); galactose metabolism (8 DEPs); fructose and mannose metabolism (6 DEPs); and alanine, aspartate, and glutamate metabolism (6 DEPs) (Figure 8C). These results suggested that photosynthesis, glycometabolism, and amino acid metabolism may be closely related to waterlogging stress in grapevine.

### Protein-Protein Interaction Analysis of Differentially Expressed Proteins

**FIGURE 9:**
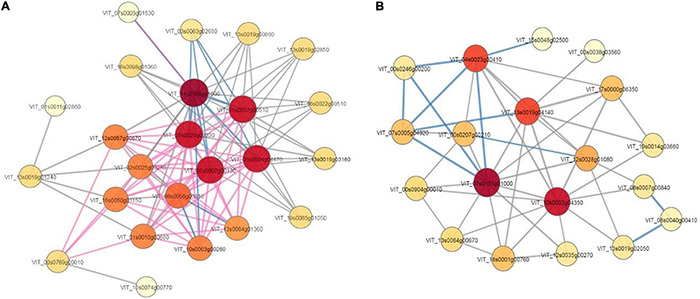
Protein-protein interaction network of differentially expressed proteins. **(A)** Proteins involved in heat shock. **(B)** Proteins involved in photosystems.

The PPI database and relevant literature were used to identify the interactions of the identified proteins or DEPs, as well as of other proteins that interacted directly with them. This PPI network (Supplementary Txts 1–3) could provide us with comprehensive information from various points of view; this information could not be determined with only a single protein analysis, and effective proteins could be found efficiently (Figure 9). According to the analysis, 13 and eight high connectivity-weight DEPs, with a degree value of more than 9, were identified in the T0d vs. T10d and T10d vs. T20d groups, respectively (Table 2). Fourteen DEPs with connectivity weights higher than 12 were identified in the T0d vs. T20d group (Table 2). To further validate direct protein-protein interactions, we selected two typical DEPs, namely, those involved in heat shock and photosystems, as constituting the PPI core (Figures 9A,B).

**TABLE 2:** DEPs with high connectivity weight in the PPI analysis between groups.

**Group**	**Protein ID**	**Weight**	**Regulated**	**Fold change**	**Description**
T0d vs. T10d	VIT_14s0108g01500	17	Up	1.46	Chaperone protein ClpB3, chloroplastic
	VIT_11s0037g00510	15	Up	1.43	Heat shock 70 kDa protein
	VIT_08s0007g00130	15	Up	1.43	Heat shock cognate 70 kDa protein 2
	VIT_06s0004g04470	15	Up	1.30	Heat shock cognate 70 kDa protein 2
	VIT_05s0020g03330	15	Up	1.46	Heat shock 70 kDa protein 8
	VIT_16s0098g00290	12	Up	1.32	Glutamate synthase 1 [NADH], chloroplastic isoform X1
	VIT_08s0056g00810	11	Down	0.75	grpE protein homolog, mitochondrial isoform X1
	VIT_10s0003g00260	10	Up	1.43	dnaJ homolog subfamily B member 13
	VIT_16s0050g01150	10	Up	1.72	Heat shock protein 83-like
	VIT_01s0010g00680	10	Up	1.50	Heat shock protein 90-1
	VIT_12s0057g00670	10	Up	1.66	Heat shock protein 90-6
	VIT_02s0025g00280	10	Up	1.46	Heat shock protein 83
	VIT_13s0064g01360	9	Up	1.35	Chaperone protein dnaJ 1, mitochondrial
T10d vs. T20d	VIT_07s0151g01000	12	Down	0.63	Photosystem I reaction center subunit II, chloroplastic
	VIT_10s0003g04350	11	Down	0.55	Photosystem I reaction center subunit psaK, chloroplastic
	VIT_14s0108g01500	11	Down	0.77	Chaperone protein ClpB3, chloroplastic
	VIT_05s0020g00600	11	Down	0.66	1-Cys peroxiredoxin
	VIT_18s0072g00340	10	Down	0.74	60S ribosomal protein L3
	VIT_19s0027g00760	10	Up	1.33	Elongation factor 2
	VIT_13s0019g04140	9	Down	0.76	Chlorophyll a-b binding protein 6, chloroplastic
	VIT_04s0023g00410	9	Down	0.76	Photosystem I reaction center subunit XI, chloroplastic
T0d vs. T20d	VIT_11s0016g01380	25	Up	1.36	40S ribosomal protein S3-3
	VIT_00s0838g00020	25	Down	0.76	30S ribosomal protein S5, chloroplastic-like
	VIT_05s0029g01200	23	Up	1.38	Methionine–tRNA ligase
	VIT_19s0027g00760	20	Up	1.34	Elongation factor 2
	VIT_14s0030g00660	16	Down	0.43	Bifunctional 3-dehydroquinate dehydratase/shikimate dehydrogenase, chloroplastic
	VIT_11s0016g03720	14	Up	1.34	Aspartate aminotransferase, cytoplasmic
	VIT_01s0010g00680	13	Up	1.40	Heat shock protein 90-1
	VIT_12s0028g03770	13	Down	0.76	DNA-directed RNA polymerase II subunit RPB2
	VIT_16s0050g01150	13	Up	1.55	Heat shock protein 83-like
	VIT_12s0035g01130	12	Up	1.31	Elongation factor 1-gamma-like
	VIT_13s0139g00170	12	Up	1.33	Uridine 5′-monophosphate synthase
	VIT_11s0016g03510	12	Down	0.72	Elongation factor Ts
	VIT_12s0057g00670	12	Up	1.59	Heat shock protein 90-6, mitochondrial
	VIT_05s0020g00600	12	Down	0.45	1-Cys peroxiredoxin

### Validation of Tandem Mass Tag Data by Quantitative Reverse-Transcription PCR

**FIGURE 10:**
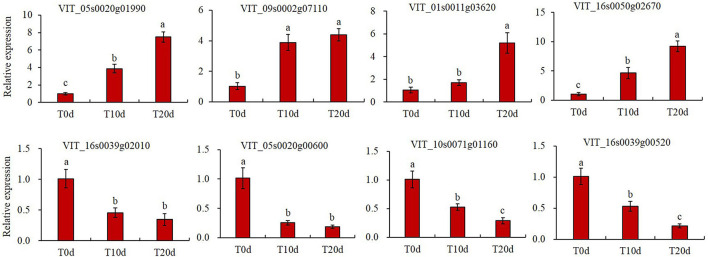
Validation of TMT data by qRT-PCR. The grapevine actin gene (AB073011) was used as an internal control. Three independent experiments were conducted for at least three biological replicates each. The error bars represent the means ± standard deviation (SDs). Columns with the same letter were not significantly different at *p* < 0.05.

To validate the data from the TMT-based proteomic profiles, we randomly selected eight DEPs to determine their relative transcript abundance by qRT-PCR. The DEPs encoded by these genes included serine/threonine protein kinase ULK4, E3 ubiquitin protein ligase, caffeoyl shikimate esterase, spermine synthase, TOM1-like protein 2, 1-Cys peroxiredoxin, ferredoxin-thioredoxin reductase and DNA-binding protein SMUBP-2. The qRT-PCR results showed that these eight genes exhibited significantly different expression in grapevine rootstock leaves after waterlogging treatment (Figure 10), and their expression trends were essentially consistent with the changes in abundance of the corresponding proteins, as revealed by the TMT technique (Supplementary Spreadsheet 1).

## Discussion

All plants require water to live, but excessive amounts of water, waterlogging or flooding results in stress and prevents gas exchange between the soil and atmosphere. Proteomics research is helpful for revealing complex changes in grapevine leaves under waterlogging stress and can provide new information concerning the grapevine response to waterlogging stress in the field. Due to the strong waterlogging resistance, SO4 grapevine rootstock plants were used as test material (Li et al., 2013b). In this study, we applied TMT labeling coupled with an LC-MS/MS approach to profile and compare the proteome of T0d, T10d, and T20d waterlogging-treated grapevine rootstock leaves. A total of 5,578 proteins were identified in the three different waterlogging-treated samples of grapevine rootstock. Comparative analysis revealed significant differences in the expression levels of proteins when T0d and T10d (214 proteins), T0d and T20d (529 proteins), and T10d and T20d (314 proteins) were compared.

### Effects of Waterlogging Stress on Reactive Oxygen Species Scavenging System-Related Proteins

To a certain extent, the plant oxidative state can reflect the ability of a plant to resist biotic or abiotic stress (Kawano, 2003; War et al., 2011). The generation and scavenging of Reactive Oxygen Species (ROS) constitute one of the plant defense mechanisms against abiotic stresses (Li et al., 2016). When plants face environmental stresses, ROS with high chemical activities are generated, which can activate plant defense mechanisms. However, excessive amounts of ROS cause great damage to biological macromolecules such as chloroplasts, mitochondria and lipids, thus affecting their normal physiological, and biochemical functions (Asada, 2006; Maffei et al., 2007; Oskuei et al., 2017). Protective enzymes, including SOD, POD, and CAT, as well as ascorbic acid and glutathione, antioxidants that play functions in scavenging ROS, are used to mitigate oxidative damage and protect cells. Ascorbic acid peroxidase (APX), whose activity is regulated by various signaling molecules, can efficiently scavenge excess H_2_O_2_ (Apel and Hirt, 2004; Bóka et al., 2007).

Previous research has shown that peroxidases play an important role in the reduction in ROS by catalyzing the redox reaction of H_2_O_2_ with various hydrogen donors (Agarwal et al., 2005). Our study also showed that the expression of some peroxidases was induced/depressed by waterlogging treatment. For example, the expression of peroxidase (VIT_07s0130g00220 and VIT_06s0004g01240) was downregulated in T10d and T20d, but the expression of cationic peroxidase (VIT_08s0058g00970) and L-ascorbate peroxidase (VIT_08s0040g03150) was upregulated in T10d and T20d compared with T0d. Moreover, the expression of glutathione peroxidase (VIT_05s0102g00120) was upregulated in T10d and downregulated in T20d compared with T0d. This may have occurred because there are many genes encoding peroxidases in plant cells, and numerous types of peroxidase play important roles in plant stress resistance (Apel and Hirt, 2004; Xu X. et al., 2018). Glutathione S-transferase (GST) is also involved in metabolism, scavenging free radicals and alleviating oxidative damage (Ge et al., 2013; Cheng et al., 2016), and this enzyme can also detoxify membrane lipid peroxides and oxidized DNA degradation products by binding to reduced glutathione (Zhang K. W. et al., 2014). Thus, increased GST (VIT_07s0005g04890) activity may contribute to grapevine resistance under waterlogging stress.

In terms of physiological indexes, the increase in MDA and H_2_O_2_ contents and the decrease in chlorophyll a and b contents show that waterlogging treatment harms grapevine rootstocks. These results are consistent with previous studies in grapevine (Li et al., 2013a, b). However, the change trends of the activity of four kinds of enzymes, SOD, POD, CAT, and APX, were different. It is worth mentioning that the change trend of SOD enzyme activity is consistent with the change trend of protein (VIT_02s0025g04830) expression. These results suggested that waterlogging stress enhances the waterlogging adaptation or resistance of grapevine leaves by improving the scavenging capacity of ROS. Similar results have been found in grapevine leaves after heat stress (Liu et al., 2014) and in maize leaves after shade stress (Gao et al., 2020). Many antioxidant enzymes have been proved to be critical for the survival of many plants under different levels of waterlogging, e.g., cucumber (Xu et al., 2016), wheat (Pan et al., 2019), and soybean (Alam et al., 2011). In these plants, the expression patterns of ROS-scavenging related proteins showed significant differential under flooded conditions. Consistent with numerous studies that have shown a correlation between the ability to ameliorate ROS and survival under different levels of waterlogging, the high induction of ROS network proteins in waterlogged grapevine showed that strong detoxification was critical for survival (Zhu et al., 2018). Moreover, there may be some additional complex mechanisms that regulate ROS scavenging system activity in addition to regulating protein expression levels.

### Effects of Waterlogging Stress on Photosynthesis-Related Proteins

Photosynthesis is known to be one of the most waterlogging-sensitive processes due to its complex mechanism and requirement for enzymes. Moreover, photosynthesis is directly related to plant productivity and energy utilization. In this study, we identified 6, 27, and 18 DEPs involved in photosynthesis, light reactions and light harvesting in photosystem I in the T0d vs. T10d, T0d vs. T20d, and T10d vs. T20d comparison groups, respectively. These accounted for 3.2, 9.6, and 16.2% of all the DEPs in each group (Supplementary Spreadsheet 3). Photosynthesis, including both the light reactions and carbon fixation reactions, determines plant productivity and energy efficiency and is very sensitive to abiotic stress (Flexas et al., 2004; Chen et al., 2016). Compared with that in T0d, the expression of chlorophyll a/b-binding proteins (VIT_19s0014g00160, VIT_07s0005g02220, VIT_17s0000g06350, VIT_19s0014g03660, and VIT_13s0019g04140) T10d and T20d was upregulated and downregulated, respectively. These results indicated that waterlogging treatment for a short time could increase the operation rate of the photosynthetic electron transport chain, which would provide sufficient restoration and adenosine triphosphate (ATP) for the waterlogging stress response. However, along with the prolonged waterlogging treatment time, the photosynthesis efficiency of grapevine progressively decreased (Li et al., 2013a, b).

Waterlogging treatment also decreased the expression of photosystem I- and photosystem II-related proteins (VIT_00s0246g00200, VIT_07s0005g04920, VIT_10s0003g04 350, VIT_07s0151g01000VIT_13s0064g00670, VIT_05s0020g03 440, VIT_07s0005g04400, VIT_12s0059g01810, and VIT_01s0 011g02150). Ferredoxin is the terminal oxidase of the photosynthetic electron transport chain, while cytochrome P450 acts as a terminal oxidase to accept NADPH electrons and jointly participates in electron transport (Cheng et al., 2016; Wu et al., 2016). Our results showed that waterlogging treatment decreased the expression ferredoxin (VIT_12s0035g00270), ferredoxin-thioredoxin reductase (VIT_10s0071g01160 and VIT_14s0066 g01900), ferredoxin-NADP reductase (VIT_18s0001g14450), cytochrome P450 (VIT_03s0167g00190 and VIT_07s0129g 00790), and cytochrome b6-f complex iron-sulfur subunit 1 (VIT_19s0014g03850) proteins, which means that waterlogging stress may affect the photosystems in grapevine by inhibiting electron transport reactions. The levels of basic metabolites such as carbon metabolites need to be adjusted to generate a new balance under abiotic stress. Altogether, these results at the proteomic and physiological level suggested that waterlogging stress in grapevine was undoubtedly closely tied to primary photosynthesis metabolic processes, and the decrease of photosynthetic activities was associated with chlorophyll loss (Zhu et al., 2018).

### Effects of Waterlogging Stress on Energy Production-Related Proteins

Plants break down complex organic matter into simple compounds via respiratory metabolism and release energy to maintain plant intermediate metabolites and energy needs (Liu et al., 2014; Zhang L. et al., 2014). Proteomics analysis showed that the expression of the polysaccharide degradation-related proteins endo-1,3;1,4-beta-D-glucanase (VIT_07s0104g00430 and VIT_07s0104g00440), alpha-glucosidase (VIT_02s0087g00030), beta-glucosidase (VIT_06s0004g01430 and VIT_05s0077g00550), and water dikinase (VIT_05s0062g00900) increased in T10d and T20d compared with T0d, indicating that these enzymes play important roles in the waterlogging tolerance of grapevine. Similar to these results, expression of alternative NAD(P)H-dependent quinone oxidoreductase-like proteins (VIT_12s0057g00250 and VIT_07s0031g02690) in the complex I of the mitochondrial respiratory chain was also greatly induced by waterlogging treatment. These increases may mean that grapevine can compensate for the lack of energy supply by enhancing electron transport and ATP synthesis in the respiratory chain. Beta-galactosidase and alcohol dehydrogenase are also important for producing energy and carbon sources (Zhuang et al., 2006; Zhang et al., 2020). The downregulated expression of beta-galactosidase (VIT_09s0002g02120, VIT_06s0004g03020, and VIT_07s0031g02480), alcohol dehydrogenase (VIT_07s0005g04610) and other proteins involved in glycolysis and the tricarboxylic acid cycle after waterlogging treatment shows that waterlogging disrupts glycolysis, the tricarboxylic acid cycle, and other energy metabolic pathways and hinders energy supplies and plant growth. Meanwhile, the alcohol dehydrogenase was also found to exhibit bidirectional functions in regulating waterlogging responses in grapevine (Zhu et al., 2018). In addition, the upregulated expression of sucrose-phosphatase 2 (VIT_12s0055g00840), phosphoenolpyruvate carboxykinase (VIT_00s1995g00010), and 1,4-alpha-glucan-branching enzyme (VIT_18s0001g00060 and VIT_19s0090g00920) also shows that the waterlogging tolerance of grapevine is closely related to energy metabolism, and the flexibility of energy metabolism may help to improve the resistance of grapevine to waterlogging stress. It is consistent with previous studies (Zhu et al., 2018; Ruperti et al., 2019).

### Effects of Waterlogging Stress on Other Defense-Related Proteins

The response of plants to waterlogging stress is a complex process. To cope with waterlogging stress, plants have developed various mechanisms to protect cellular activities and maintain integrity (Pucciariello et al., 2019; Garcia et al., 2020). In addition to the above proteins, other defense-related proteins have also been identified, including serine/threonine protein kinases, zinc-finger proteins, and heat-shock proteins. Serine/threonine protein kinases compose a large protein family and play key roles in many signal transduction processes, including plant stress signaling and responses (Lease et al., 2001; Lee and Kim, 2015; Monika et al., 2018). This study showed that waterlogging stress contributes to the expression of serine/threonine protein kinases (VIT_07s0031g03210, VIT_07s0191g00070, and VIT_05s0020g01990), which showed that these proteins may be related to grapevine tolerance to waterlogging.

Protein metabolic processes are closely associated with plant growth and development via their regulation of a series of biological activities (Swidzinski et al., 2004). Zinc-finger proteins are transcriptional regulatory factors that regulate gene expression through their zinc and iron centers binding to DNA (Klug, 2010; Zhang et al., 2016), which in turn modulates plant growth and development by regulating gene expression (Laity et al., 2001; Chen et al., 2013). Zing-finger proteins also play a very important role in plant responses to biotic and abiotic stresses (Huang et al., 2009; Yue et al., 2015; Wang et al., 2016; Zhang et al., 2016). In rice, upregulated expression of two zinc-finger proteins (Q67TK9 and Q10N88) and downregulated expression of another zinc-finger protein (Q5YLY5) contribute to increased heat tolerance duration at night during grain filling. Our results showed that the expression of zinc-finger superfamily proteins (VIT_01s0011g01690, VIT_13s0156g00320, VIT_11s0016g05750, and VIT_16s0098g00360) increased in the T10d and T20d treatments, which might be related to the waterlogging tolerance of grapevine.

Most proteins are required to fold into specific three-dimensional structures to perform their functional activity. However, many proteins fold aberrantly and even aggregate under stress conditions (Hartl et al., 2011; Kim et al., 2013). Aberrant protein folding and aggregation occur with increasing frequency under environmental stresses such as high temperature and drought (Dobson, 2003). Therefore, plants have evolved numerous mechanisms to address aberrant folding and aggregation. Heat shock proteins (HSPs) have been demonstrated to have functions in preventing aggregation and promoting efficient folding under adverse conditions (Jakob and Buchner, 1994). Recent research has also demonstrated that drought causes a marked increase in the expression of several small HSPs, and waterlogging inhibits the expression of some HSPs in maize leaves (Yang et al., 2014; Hu et al., 2020). In our study, a total of 14 differentially expressed HSPs (VIT_13s0019g03160, VIT_02s0025g00280, VIT_16s0098g01060, VIT_13s0019g03090, VIT_13s0019g03000, VIT_13s0019g02850, VIT_12s0028g01390, VIT_16s0050g01150, VIT_13s0019g00860, VIT_12s0057g00670, VIT_01s0010g00680, VIT_16s0022g00510, VIT_01s0010g02290, and VIT_19s0085g01050) were identified after waterlogging treatment. It is worth noting that the expression of all 14 differentially expressed HSPs was significantly upregulated in T10d compared to T0d, but with increasing waterlogging treatment time, the expression of all 14 differentially expressed HSPs was downregulated in T20d compared to T10d. However, the expression of the key constituents of the degradation system, 26S proteasome subunits (VIT_07s0031g01970 and VIT_00s0215g00050), was upregulated in waterlogged plants. In addition, the abundance of proteins associated with phosphatase (VIT_12s0028g03310 and VIT_16s0098g01650) decreased greatly in T10d and T20d. These results suggested that waterlogging affected protein folding and other processing-related processes, which impeded the proteins from performing their functions.

## Conclusion

In the present study, we used a TMT-based LC-MS/MS technique to compare the abundance of proteins among three different waterlogging-treated grapevine rootstock samples (T0d, T10d, and T20d). Our results indicated that waterlogging triggered notable changes in defense system activity by modulating the expression of ROS-, photosynthesis-, energy production-, and other defense-related proteins. Integrated analysis of proteomics in response to waterlogging of grapevine can help provide an in-depth understanding of various physiological and biochemical mechanisms, provide a scientific basis to explore the mechanism underlying grapevine waterlogging resistance, and lay a foundation for further functional exploration and verification of related genes and proteins. In summary, although the sessile nature of plants makes them vulnerable to various kinds of biotic and abiotic stresses, plants have evolved sophisticated mechanisms to recognize and respond to these stresses.

## Data Availability Statement

The original contributions presented in the study are publicly available. This data can be found here: Proteome Xchange Consortium database under accession number PXD027839.

## Author Contributions

XW, WW, and ZW conceived the research. XW, YQ, BW, and LY designed the experiments. XW carried out all the experiments, analyzed the data, and wrote the article, with contributions from all the authors. All authors contributed to the article and approved the submitted version.

## Conflict of Interest

The authors declare that the research was conducted in the absence of any commercial or financial relationships that could be construed as a potential conflict of interest.

## Publisher’s Note

All claims expressed in this article are solely those of the authors and do not necessarily represent those of their affiliated organizations, or those of the publisher, the editors and the reviewers. Any product that may be evaluated in this article, or claim that may be made by its manufacturer, is not guaranteed or endorsed by the publisher.

## Footnotes

1
https://bioinfogp.cnb.csic.es/tools/venny/

2
https://cluster2.software.informer.com/3.0/

3
http://www.ebi.ac.uk/GOA/

4
http://www.ebi.ac.uk/interpro/

5
http://www.kegg.jp/kegg/

6
http://string-db.org/

7
http://www.cytoscape.org/release_notes_3_2_1.html

## Supplementary Material

Supplementary Figure 1 Pearson’s correlations of quantitation among treatments.

Supplementary Figure 2 GO analysis of DEPs in the T0d vs. T10d comparison group.

Supplementary Figure 3 GO analysis of DEPs in the T10d vs. T20d comparison group.

Supplementary Figure 4 GO analysis of DEPs in the T0d vs. T20d comparison group.

Supplementary Table 1 Primer sequences of DEP-encoding genes used for qRT-PCR.

Supplementary Spreadsheet 1 Differentially expressed proteins identified in grapevine rootstock leaves.

Supplementary Spreadsheet 2 Details of all the differentially expressed proteins.

Supplementary Spreadsheet 3 Details of the GO enrichment analysis of DEPs among the various comparison groups.

Supplementary Spreadsheet 4 Statistics of GO annotations of DEPs among the various comparison groups.

Supplementary Spreadsheet 5 Details of KEGG pathway enrichment analysis of DEPs among the various comparison groups.

Supplementary Txt 1 PPI analysis of the connectivity degree in the T0d vs. T10d comparison group.

Supplementary Txt 2 PPI analysis of the connectivity degree in the T10d vs. T20d comparison group.

Supplementary Txt 3 PPI analysis of the connectivity degree in the T0d vs. T20d comparison group.
