# Supplementary figures and images for: Comparative Proteomic Analysis of Grapevine Rootstock in Response to Waterlogging Stress
Source: Front Plant Sci. 2021 Oct 29;12:749184. doi: 10.3389/fpls.2021.749184 (PMC8589030; doi:10.3389/fpls.2021.749184)

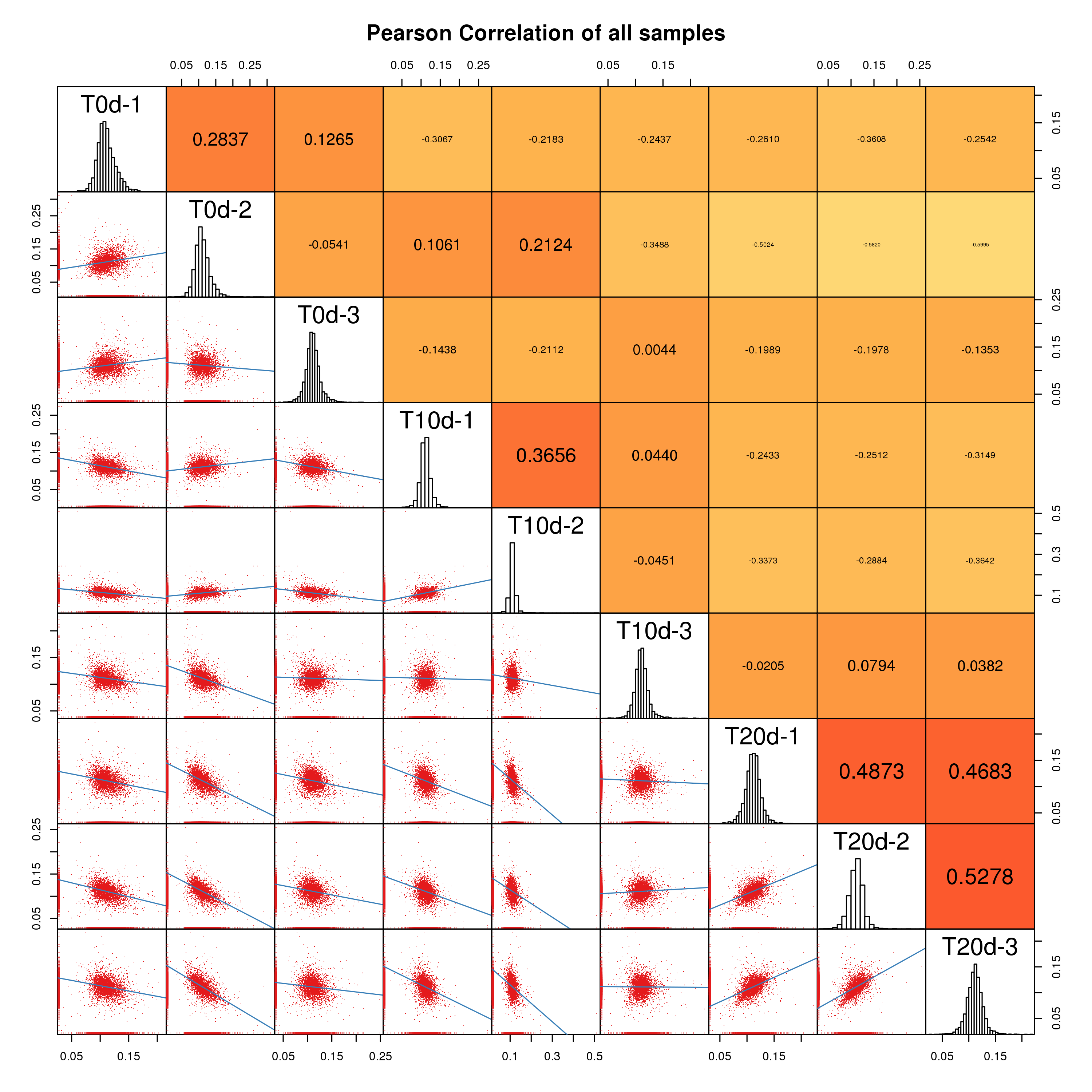

Supplement: Supplementary Figure 1 — Pearson’s correlations of quantitation among treatments. [file Data_Sheet_1.ZIP › Supplementary Figure S1.png]

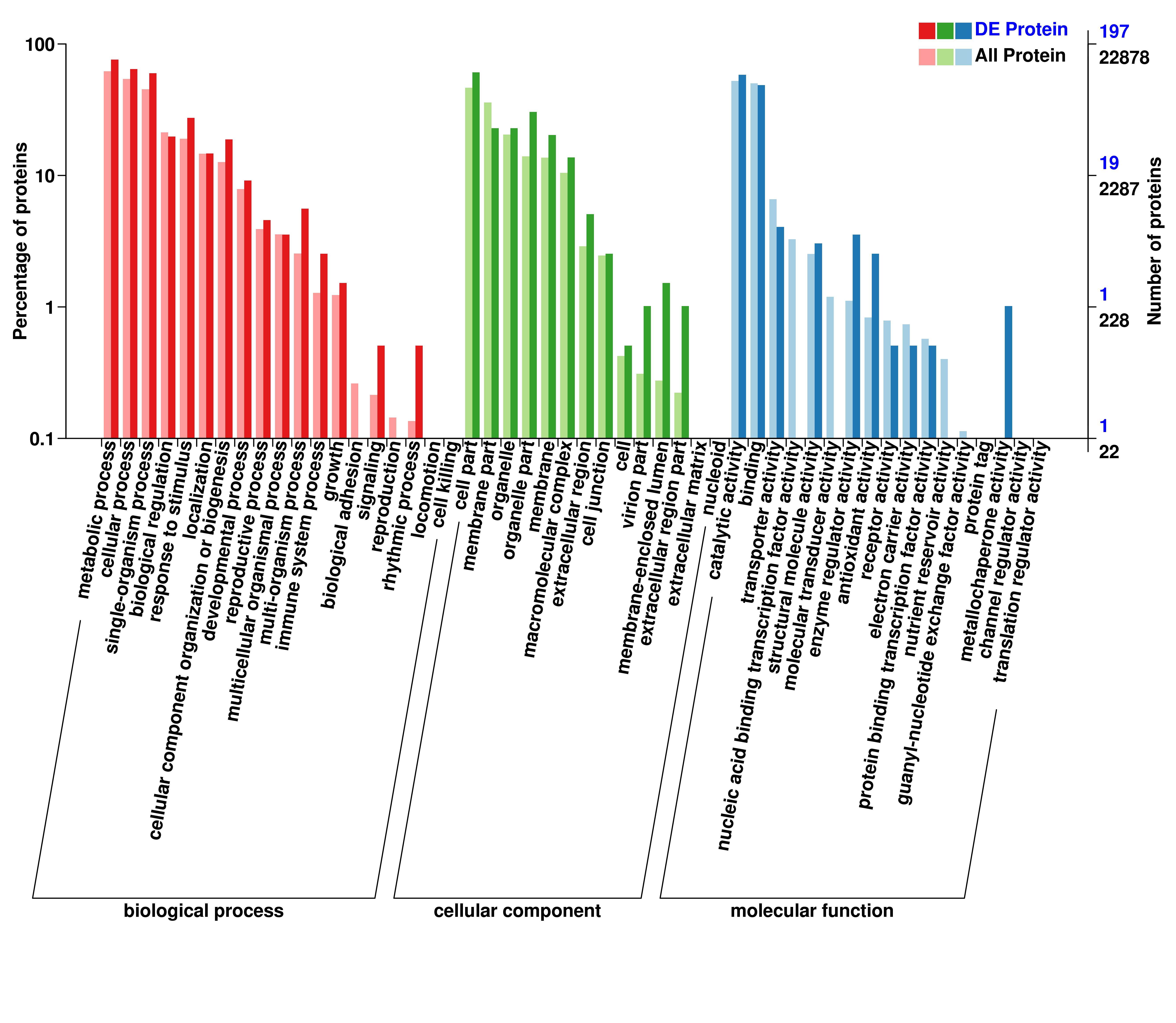

Supplement: Supplementary Figure 1 — Pearson’s correlations of quantitation among treatments. [file Data_Sheet_1.ZIP › Supplementary Figure S2.png]

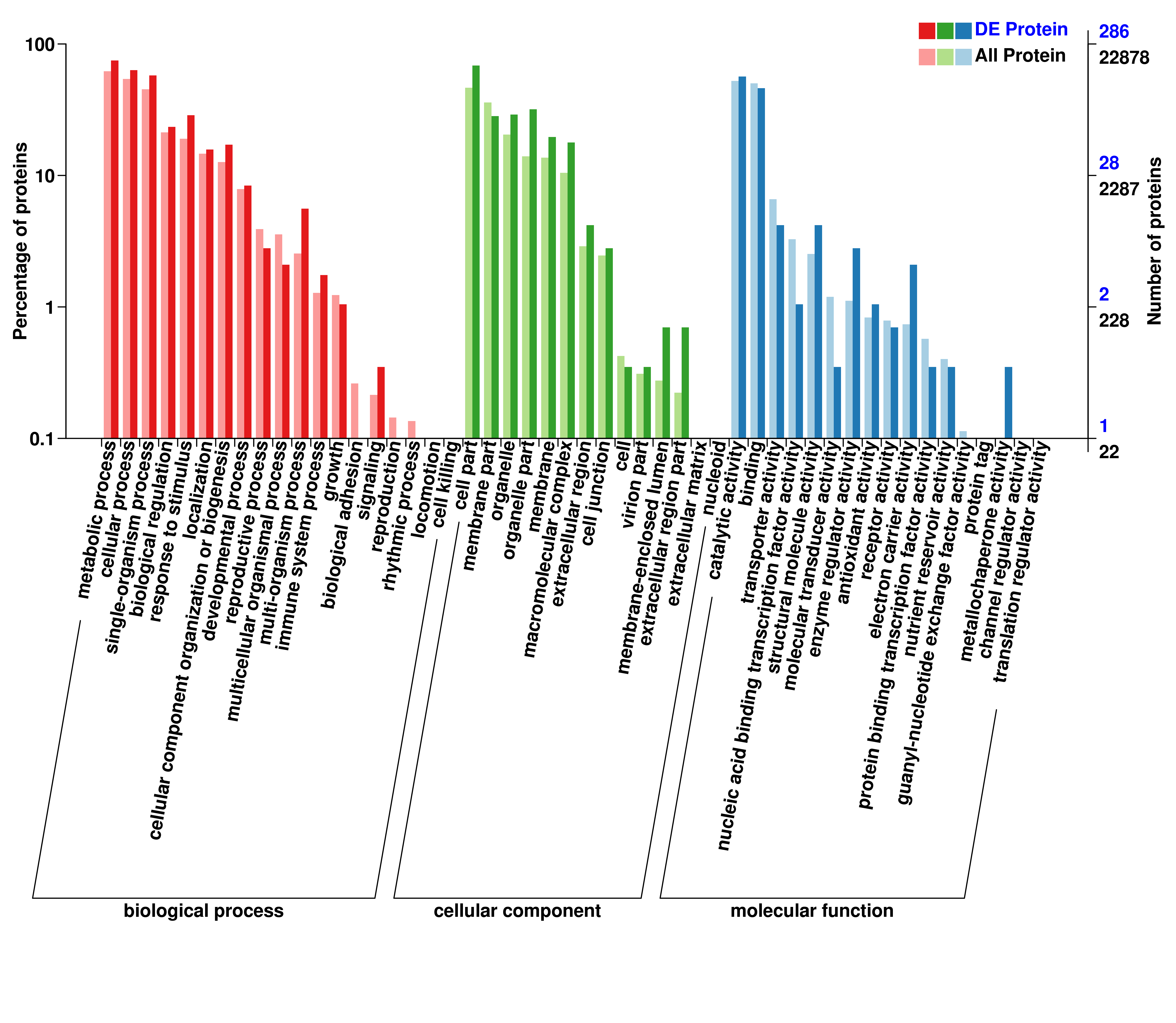

Supplement: Supplementary Figure 1 — Pearson’s correlations of quantitation among treatments. [file Data_Sheet_1.ZIP › Supplementary Figure S3.png]

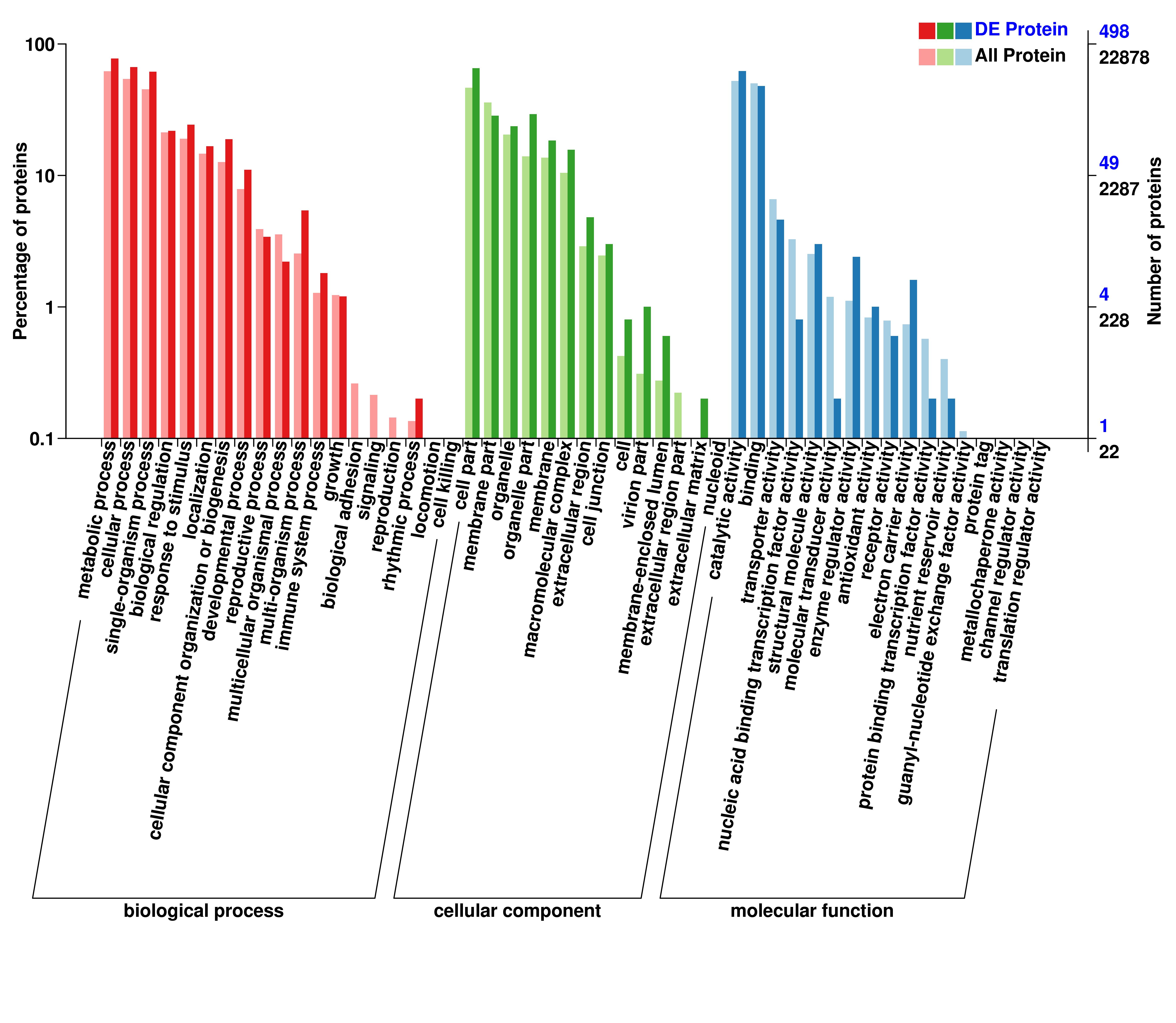

Supplement: Supplementary Figure 1 — Pearson’s correlations of quantitation among treatments. [file Data_Sheet_1.ZIP › Supplementary Figure S4.png]
