# Supplementary material for: Comparative Proteomic Analysis of Grapevine Rootstock in Response to Waterlogging Stress
Source: Front Plant Sci. 2021 Oct 29;12:749184. doi: 10.3389/fpls.2021.749184 (PMC8589030; doi:10.3389/fpls.2021.749184)
Supplement: Supplementary Figure 1 — Pearson’s correlations of quantitation among treatments. [file Data_Sheet_1.ZIP › Supplementary Table S1.doc]

**Table S1** Primers used for qRT- PCR

| Gene ID | Protein description | Forward sequence (5’to 3’) | Reverse sequence (5’to 3’) |
| --- | --- | --- | --- |
| VIT_05s0020g01990 | Serine/threonine-protein kinase ULK4 | CTGGTTGGCAGGTTTCAAAT | GTTCCCTCCCTGACTGCATA |
| VIT_09s0002g07110 | E3 ubiquitin-protein ligase | ATAGCCGTGGCTACACATCC | ACCAACCACAGTCCCTTCTG |
| VIT_01s0011g03620 | Caffeoylshikimate esterase | AGAAGGTTTCATCGCCATTG | CCCTTCCAGAATGCAGTGAT |
| VIT_16s0050g02670 | Spermine synthase | CGTCAGCATATGGCAAAGTG | TGAACAAAGGGGAAGGTGAG |
| VIT_16s0039g02010 | TOM1-like protein 2 | GAAAGTTTGGCCCCCATATT | GCCACATCAAATGCTTCCTT |
| VIT_05s0020g00600 | 1-Cys peroxiredoxin | CAGTCCCACAAGGAGTGGAT | ATCCGGATCCACCATGTTTA |
| VIT_10s0071g01160 | Ferredoxin-thioredoxin reductase | TGTGAAATCCCCTTCCTCTG | TTGTTCAAAGGCTGCTGATG |
| VIT_16s0039g00520 | DNA-binding protein SMUBP-2 | GCAAGGTATCTCAGCCGAAG | AGCTCAGACATCCGCTTTGT |
| AB073011 | Actin | GGAAGCTGCGGGA ATTCATGAG | CCTTGATCTTCATGCTGCTGGG |
